# Automated Management of Exercise Intervention at the Point of Care: Application of a Web-Based Leg Training System

**DOI:** 10.2196/rehab.4812

**Published:** 2015-11-23

**Authors:** Vadim N Dedov, Irina V Dedova

**Affiliations:** ^1^MedExercise ProjectResearch and DevelopmentMDXD Pty LtdSydneyAustralia; ^2^Department of AnatomySchool of Medical SciencesUniversity of New South WalesSydneyAustralia

**Keywords:** digital intervention, exercise intervention, cardiac rehabilitation, training equipment, online monitoring, exercise dose, telerehabilitation, leg mobilization, Web-based apps, eHealth recordings

## Abstract

**Background:**

Recent advances in information and communication technology have prompted development of Web-based health tools to promote physical activity, the key component of cardiac rehabilitation and chronic disease management. Mobile apps can facilitate behavioral changes and help in exercise monitoring, although actual training usually takes place away from the point of care in specialized gyms or outdoors. Daily participation in conventional physical activities is expensive, time consuming, and mostly relies on self-management abilities of patients who are typically aged, overweight, and unfit. Facilitation of sustained exercise training at the point of care might improve patient engagement in cardiac rehabilitation.

**Objective:**

In this study we aimed to test the feasibility of execution and automatic monitoring of several exercise regimens on-site using a Web-enabled leg training system.

**Methods:**

The MedExercise leg rehabilitation machine was equipped with wireless temperature sensors in order to monitor its usage by the rise of temperature in the resistance unit (Δ*t*°). Personal electronic devices such as laptop computers were fitted with wireless gateways and relevant software was installed to monitor the usage of training machines. Cloud-based software allowed monitoring of participant training over the Internet. Seven healthy participants applied the system at various locations with training protocols typically used in cardiac rehabilitation. The heart rates were measured by fingertip pulse oximeters.

**Results:**

Exercising in home chairs, in bed, and under an office desk was made feasible and resulted in an intensity-dependent increase of participants’ heart rates and Δ*t*° in training machine temperatures. Participants self-controlled their activities on smart devices, while a supervisor monitored them over the Internet. Individual Δ*t*° reached during 30 minutes of moderate-intensity continuous training averaged 7.8°C (SD 1.6). These Δ*t*° were used as personalized daily doses of exercise with automatic email alerts sent upon achieving them. During 1-week training at home, automatic notifications were received on 4.4 days (SD 1.8). Although the high intensity interval training regimen was feasible on-site, it was difficult for self- and remote management. Opportunistic leg exercise under the desk, while working with a computer, and training in bed while viewing television were less intensive than dosed exercise bouts, but allowed prolonged leg mobilization of 73.7 minutes/day (SD 29.7).

**Conclusions:**

This study demonstrated the feasibility of self-control exercise training on-site, which was accompanied by online monitoring, electronic recording, personalization of exercise doses, and automatic reporting of adherence. The results suggest that this technology and its applications are useful for the delivery of Web-based exercise rehabilitation and cardiac training programs at the point of care.

## Introduction

Substantial evidence has established the value of physical activity and strongly supports the routine prescription of exercise training to all patients, including those with cardiovascular disease and other chronic diseases [1]. However, the current framework of cardiac rehabilitation (CR) is not sustainable due to significant barriers such as high costs [2]. Modern technology facilitates the development of digital interventions for health care, which can provide effective and potentially cost-effective models for improving patient engagement and health outcomes [3]. However, despite a large number of physical activity apps on the market, there is a shortage of evidence-based apps that can be used clinically [4,5].

**Figure 1:**
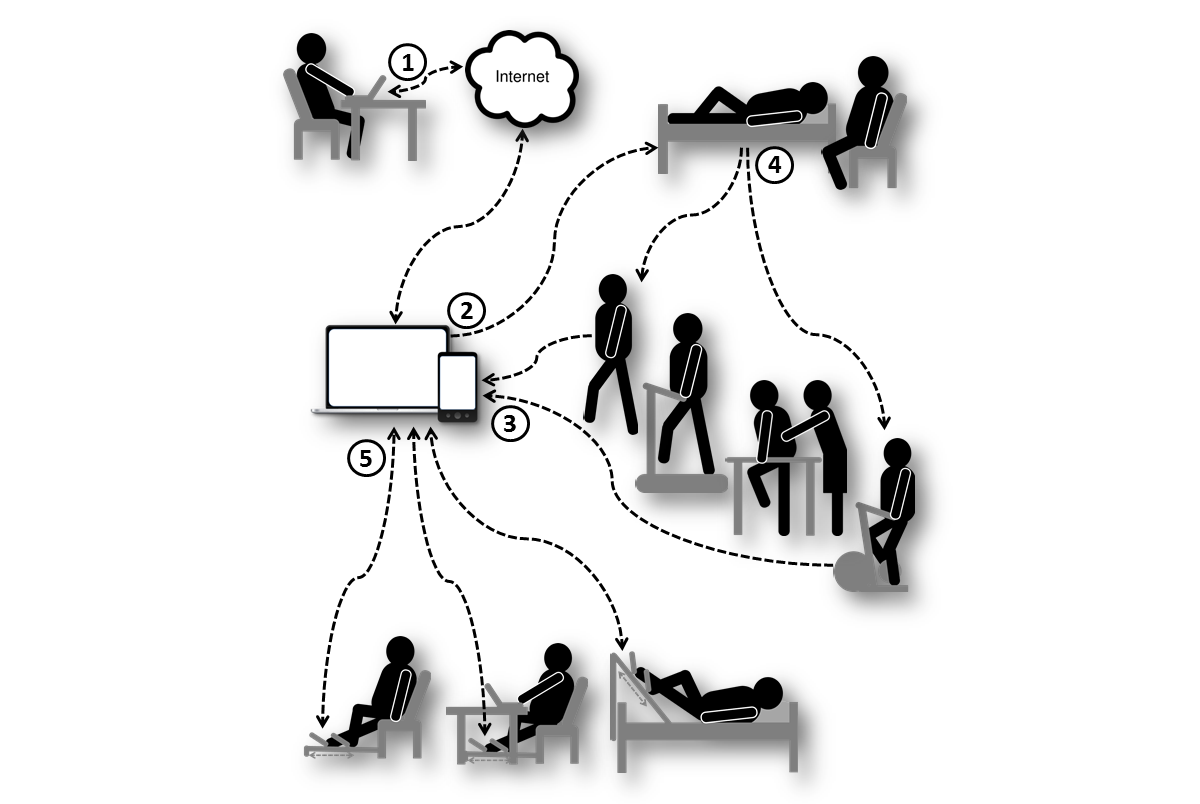
CR delivery framework using modern technology, where arrows indicate: (1) remote management by healthcare providers; (2) digital intervention delivery to target populations; (3) physical activity monitoring; (4) conventional exercise and cardiac rehabilitation; and (5) on-site training with the MedExercise system in different settings.

The overall contribution of technology in improving CR delivery [6] can be conceptualized in relation to four essential components of CR: patient management, motivation, actual training and monitoring (Figure 1). The process of rehabilitation is normally guided by relevant health care providers such as physiotherapists, who perform patient assessments, prescribe training programs, assist in training, and control patient compliance. Figure 1 shows that modern telehealth technology allows remote patient management (arrow 1), including telecare of chronic diseases at home environment [7]. Provision of CR using telehealth approaches was found not significantly inferior to the center-based programs in patients with cardiovascular diseases [8].

Arrow 2 demonstrates that behavioral interventions such as physical activity promotion can be efficiently delivered to the patients using electronic means. The major advantage of digital interventions is the capacity for patient-centered approaches [9] such as age-appropriate apps for older adults [10]. Web-based programs have been suggested as an alternative method of CR [11] and mobile technology is considered a valuable approach to improving access to CR [12]. Nevertheless, access to health information by itself may have insufficient impact on the health-related behavior in patients with cardiovascular diseases [13].

Arrow 3 indicates that modern technology also allows telemonitoring of exercise training by measuring physiological parameters and exercise volumes. For example, the feasibility of home-based telemonitoring in patients with heart failure has been demonstrated in several studies [14]. Wireless multibiosensor systems were found valid for real-time tracking of physical activity among cardiac patients [15] and there are a large number of inexpensive physical activity counters for use in clinical and public health settings [16]. However, accelerometers and pedometers have significant limitations [17] so that subjective self-reporting still remains the main method of physical activity surveillance [18].

Arrow 4 exemplifies typical methods of patient training, where CR can be delivered in self-control, face-to-face or group-based settings. This model has not changed over the past two decades [19] and methods of endurance training such as running and cycling are the same as used by the healthy fit people [6,20]. Because diseases, obesity, and aging reduce patients’ endurance and mobility [21,22], their low exercise capacity can be considered as a barrier to sustained CR. Other barriers include lack of transport, financial cost, and embarrassment about participation [23]. Many patients have to organize and support their daily training by themselves, including travel to the gym and expensive professional assistance. The higher costs and resource intensity make them disadvantaged in their access to exercise training compared with the healthy population [19].

Figure 1 shows that the phase of actual training has been influenced by technology to a lesser extent than other components of CR. It could be suggested that taking patients away from the point of care for training and use of conventional exercise methods may hold back implementing the full potential of technology in CR. Therefore, we have developed a Web-enabled system for training on-site, which consisted of an innovative leg rehabilitation system integrated with mobile devices (arrows 5). This study demonstrated that typical exercise regimens used in CR were feasible at home with remote monitoring and automated reporting of compliance. Application of this technology might be useful for the integration of exercise training with digital interventions at the point of care to improve sustainability of CR.

## Methods

### Equipment and Data Collection

MedExercise ST01 leg training system (MDXD Pty Ltd) was used in this work. This portable system has been developed for exercise rehabilitation on-site and consists of a variable resistance unit with two pedals, means for attachment to furniture, and a measurement module as detailed elsewhere [24,25]. In addition, the resistance units were equipped with wireless temperature sensors (Monnit) so that temperature was measured in 1-minute intervals, transmitted to electronic devices using MonnitLink USB gateways and processed using iMonnit stand-alone or cloud-based software. Before each training session, the temperature values were set to zero. Exercise-induced rise of temperature in the resistance unit (Δ*t*°) was used to monitor training activities of participants and calculate leg work output during exercise bouts. Participants’ heart rates (HRs), expressed as beat per minute (BPM), and blood oxygen saturation were monitored using fingertip pulse-oximeter CMS-50E and respective software (Contec). Blood pressure was measured using the Omron blood pressure monitor HEM7200 (Omron Healthcare). Data were analyzed using Excel spreadsheet software (Microsoft).

### Study Design

Arrows 5 (Figure 1) illustrate the experimental setups tested in this study, which represent the possible on-site locations of CR including standard upright chairs, recumbent armchairs, beds, and office desks. The participants trained with MedExercise devices attached to the pieces of furniture they normally used at home. Standard electronics such as laptop were equipped with wireless USB gateways and used by participants for monitoring of self-control training. When devices were connected to the Internet, the supervisor could remotely monitor training of participants in real time and access Δ*t*° data, which were continuously recorded for retrospective analysis.

The following 5 CR protocols were tested: (1) moderate-intensity continuous training (MICT), where the participants exercised at a steady-state intensity for specified durations, which ranged from 10 to 120 minutes; (2) dosed MICT protocol, where participants were training at the HR of 100 ± 10 BPM for 30 minutes to match the recommended daily exercise volume for healthy adults [20]; (3) high-intensity intermittent training, using a modified Wingate protocol [26], which included 10 cycles of alternate exercise at high (30 seconds) and moderate (2 minutes) intensities; (4) concurrent training at the office desk while participants were working with computers; and (5) training in the bed, where recumbent participants were exercising ad libitum, while viewing TV or entertaining themselves with their electronic devices.

### Participants

The inclusion criteria were (1) absence of known medical contraindications to regular exercise training; (2) capacity for installation of training equipment and its regular use at home; and (3) connection to the Internet and ability to manage electronic data. The reasons for exclusion included (1) inability to provide informed consent; (2) not adhering to the prescribed training regimen; and (3) failure to use technology reliably. Because this study was focused on the validation of new technology, recruitment was limited to the needs for testing of various training programs in different settings. Overall 7 eligible volunteers (25-52 years old) participated from 2013 to 2015.

## Results

### Various Training Regimens Feasible On-Site

**Figure 2:**
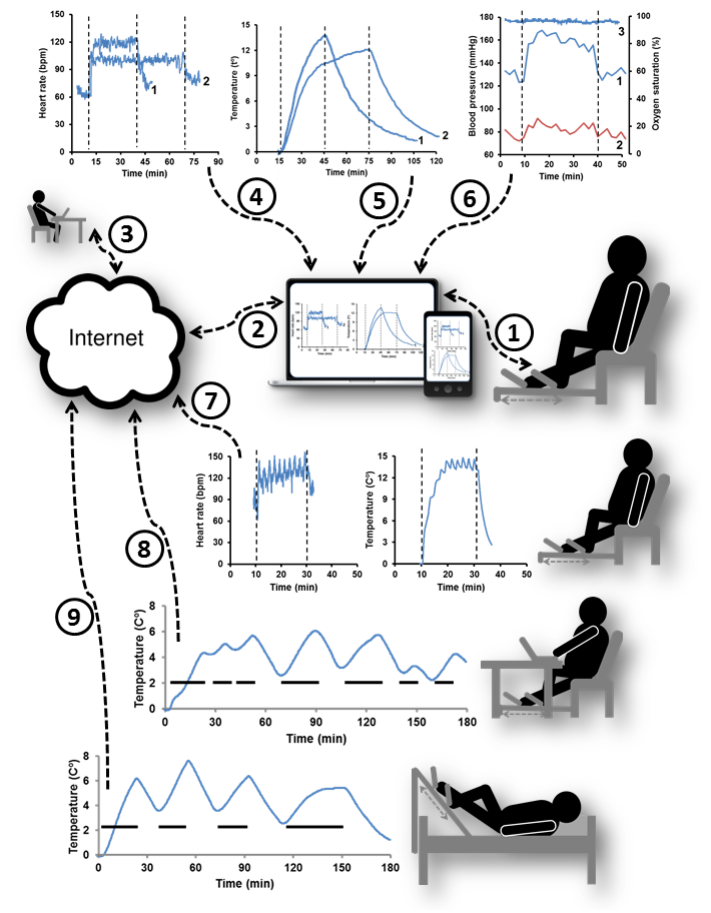
Typical settings and data recorded during this study, where arrows indicate: (1) wireless data transfer between the participant and electronic device; (2) connection to the Internet; (3) remote access by supervisor; parallel (4) HR and (5) Δt° recordings during exercise at vigorous and moderate intensities indicated by curves 1 and 2, respectively; (6) changes in systolic and diastolic blood pressure, and blood oxygen saturation during MICT, curves 1-3 respectively; (7) changes in HR and Δt° during high intensity interval training; changes of Δt° during (8) under-desk and (9) in-bed training. Dashes show periods of active training.

**Figure 3:**
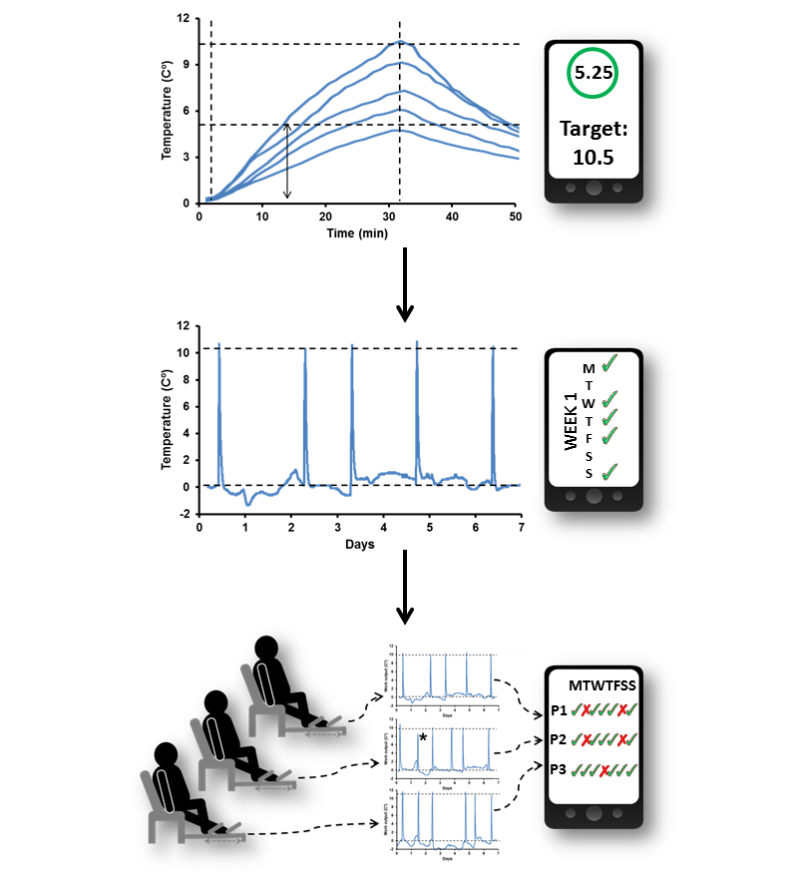
Personalization and automation of MICT doses, showing: (top row) variability of Δ*t*° produced at the same HR of 100±10 bpm and numeric expression of the MICT dose; (middle row) continuous Δ*t*° recording for 1 week and a corresponding digital interface, marking compliant days by ticks; (bottom row) data flow during this study from the home-based training systems to supervisor’s interface, reflecting daily adherence to the personalized MICT doses of multiple participant as ticks and crosses.

In this work, we first tested the feasibility of CR regimens on-site and then validated a new method for automated monitoring of compliance (Figures 2 and 3, respectively). Arrows 1-3 in Figure 2 illustrate the data flow from the training equipment to electronic devices and then over the Internet to a remote supervisor. A mobile device at hand allowed participants to monitor and adjust their own training activities in real time, while multiuser cloud-based software enabled the supervisor to access the data from multiple participants regardless of their location. Previously, we demonstrated that remote analysis of recorded data from the preceding week was useful in advising the participants on adjusting their training activities [25].

Arrows 4 and 5 point to the actual recordings of HR and Δ*t*° during exercise bouts, where Δ*t*° reflects the leg work output [24] and HR is the standard characteristic of exercise intensity [27]. It demonstrates a direct correlation between the intensity of training and participant’s leg work output because vigorous exercising at 120 ± 10 BPM resulted at a higher work output per minute indicated by the sharper rise of Δ*t*° (curve 1). However, a moderate intensity of training at 100 ± 10 BPM allowed longer durations of exercise sessions such as a 60-minute bout illustrated by Curves 2. MICT bouts of up to 120 minutes were recorded during this study (data not shown). Arrow 6 marks data input from blood pressure monitor, indicating the expected rise of systolic blood pressure during MICT [28]. The oxygen saturation of the blood remained a steady state (Curve 3).

Arrow 7 exemplifies input of HR and Δ*t*° data during high-intensity interval training. It shows the association between HR and leg work output, where the busts of intensive training caused sharp rising of HR and Δ*t*° followed by recovery periods. The feasibility of high-intensity interval training has been tested because it was shown to increase cardiorespiratory fitness by almost double that of MICT in the patients with lifestyle-induced chronic diseases [29]. However, participants found this regimen more difficult to adhere than MICT so it was not used routinely. The MICT and high-intensity interval training protocols can be described as dedicated training regimens because they have controlled exercise intensity and limited bout durations.

Nevertheless, there are real-life situations when dedicated training bouts are not feasible on a regular basis due to poor participant health or other circumstances. Consequently, we have tested an alternative exercise protocol where leg training was secondary to the sedentary activity of participant such as viewing TV. This exercise regimen can be described as opportunistic as it does not have a specified training intensity and duration. Arrows 8 and 9 indicate the leg work output of participants working with computer at the desk or lying in bed while watching TV. These recordings show that opportunistic exercise sessions had variable leg work output and overall lower training intensity than in dedicated exercise bouts. Typically, they also included breaks, but allowed prolonged leg mobilization averaging at 73.7 minutes (SD 29.7) without committing extra time to dedicated training activities. According to the participants, concurrent training reduced boredom of otherwise monotonous exercising.

### Personalization and Automation of Exercise Doses

Figure 3 explains the method of personalizing MICT doses and automated notification of compliance. The dose of daily exercise was based on the current guideline of *moderate-intensity aerobic (endurance) physical activity for a minimum of 30 minutes on 5 days each week* [6,20]. It was proposed that Δ*t*° achieved by MICT in 30 minutes would indicate a participant’s personalized dose of exercise to be taken on a daily basis. The curves at a top row demonstrate that Δ*t*° values produced by participants in 30-minute MICT bouts ranged from 4.5 to 10.5°C averaging 7.8°C (SD 1.6). These values were used as the numerical expression of personalized exercise doses as exemplified by digital interface. For example, horizontal dashed lines define a half (arrow) and the full dose of 10.5°C at the top curve so that a number 5.25°C indicates 50% of the dose. Reaching a target of 10.5°C signified taking a full personalized dose of MICT.

The participant was instructed to reach the MICT dose of 10.5°C on a daily basis. A middle curve at Figure 3 exemplifies weekly Δ*t*° recording, where each peak represents one training bout such as at the top row, but on the scale of 1 week. Once the dose of 10.5°C was achieved (dashed line), an automatic notification will be sent by email. Each message was counted as ticks on the digital interface, which demonstrates that during this week MICT doses were taken on Days 1, 3, 4, 5, and 7. Because the doses were taken on 5 days during the week, the participant was considered to have matched the recommended volume of weekly exercise [20]. A bottom row at Figure 3 shows the overall data flow allowing assessment of compliance using cloud-based software. The digital interface exemplifies that 2 participants took daily exercise doses on 5 days and one on 6 days during the week. Therefore, all 3 participants met the recommended weekly exercise doses [20].

The individual Δ*t*° averaged 7.8°C (SD 1.6) was prescribed to all participants as daily MICT doses and set as the threshold for email alerts. Exercise devices integrated with personal electronic devices were provided for training at home for 1 week. Every time Δ*t*° reached the individual threshold, automatic notifications were sent to the participants and supervisor to notify about taking the MICT dose. Overall, the automatic notifications were received on 4.4 days per week (SD 1.8). Retrospective analysis of Δ*t*° recordings allowed detection of uncompleted MICT bouts, as marked by the asterisk at the middle curve of the bottom row. Previously, we demonstrated that weekly training patterns could vary and poor adherence prompted intervention by supervisor over the phone [25].

## Discussion

### Principal Findings

In this work, we demonstrated the feasibility of delivering several exercise regimens on-site using an innovative training setup that provided the participants with integrated digital and physical interfaces. A standard mobile device such as a laptop was used as the user interface for monitoring of self-control training, whereas a portable leg rehabilitation system served as the physical interface providing means of resistance training [24]. Because data from the exercise device were inputted in real time, it operated in the capacity of a peripheral appliance to the mobile device at hand. Connection to the Internet and application of cloud-based software enabled online networking and remote monitoring of participants’ training. Future development of this system might include its integration with patient portals and activity-centered gamified apps [30].

MICT regimens such as bouts of running or cycling are commonly used in CR [6]. An advantage of the MICT mode is measurability of exercise volumes using metabolic equivalents of various physical activities as a function of time [31]. In this work, we proposed a new method for exercise quantification by measuring Δ*t*° in the training device. Because Δ*t*° correlated with exercise intensity and duration, it was proportional to the energy expenditure of the user. Therefore, Δ*t*° produced by MICT in 30 minutes could reflect the recommended amount of daily energy expenditure [6,20]. The numerical nature of Δ*t*° allowed expression of daily exercise volumes as single numbers, making it similar to the doses of drugs to be taken daily. The terminology of exercise “dose” and “dose taking” used in this study was analogous to previously proposed expressions such as exercise “pill” and “pill taking” [2,6].

### Implications for CR

The feasibility of different training regimens suggests self-sufficiency of compact and inexpensive MedExercise systems, which might allow exercise intervention without taking the patient away from the point of care (Figure 1). By contrast, the current model of CR is limited to specialized gyms because fitness machines such as treadmills and cycle ergometers are too heavy, bulky, and costly for use on-site. For example, a Motomed cycling machine, which is modified for use at the point of care, was found useable [32], but sophisticated mechanics makes it too expensive for widespread applications in CR. Motomed also lacks the networkability for remote management. Facilitating on-site training might be useful to mitigate barriers to sustained delivery of CR such as the need for daily transportation and face-to-face training of weak sedentary patients [2].

The variability of feasible exercise regimens demonstrated in this study allows customization of on-site exercise programs to provide patient-centered intervention. Depending on the level of fitness and other circumstances, patients could be offered training regimens ranging from intensive exercise bouts for relatively fit participants to extensive opportunistic training in bedridden patients. There might be many categories of patients to benefit from on-site CR, including critically ill patients, in which early leg mobilization can improve outcomes [33]. Nonclinical uses of on-site exercising include contemporary workplaces, where there is a high potential for increasing energy expenditure in sedentary workers [34]. Reduced boredom of concurrent exercise may help to improve sustainability of CR.

A single-number exercise dose introduced in this study allowed automation of compliance reporting using the digital threshold settings. This approach can simplify the long-term compliance monitoring, because a “good” adherence could be easily quantified as taking 5 or more daily MICT doses per week. Noncompliance became obvious as less than 5 alerts received in a week [6,20]. Cloud-based software can serve as a Web-based platform for health care providers, useful for controlling CR in the participant network, including remote patients at the point of care. Web-based management is likely to reduce the financial cost of services by eliminating geographical barriers and reducing labor intensity of CR per patient [8].

### Limitations

The limitations of this study include participation of only a small number of healthy volunteers and relatively short duration of intervention, which was due to the innovative nature of the technology and focus on testing new training protocols before offering them for the clinical trials. Therefore, this work should be considered as a pilot study, validating the technical feasibility of innovative technology. The encouraging results of this study warrant further research to establish the efficacy, effectiveness, and feasibility of this approach in patients with chronic diseases and other medical conditions preventable and treatable by regular exercise training.

## Abbreviations

BPM: beats per minute
CR: cardiac rehabilitation
HR: heart rate
MICT: moderate-intensity continuous training
